# Cytotoxicity and Genotoxicity of Azobenzene-Based Polymeric Nanocarriers for Phototriggered Drug Release and Biomedical Applications

**DOI:** 10.3390/polym14153119

**Published:** 2022-07-31

**Authors:** Maritza Londoño-Berrío, Sandra Pérez-Buitrago, Isabel Cristina Ortiz-Trujillo, Lina M. Hoyos-Palacio, Luz Yaneth Orozco, Lucelly López, Diana G. Zárate-Triviño, John A. Capobianco, Pedro Mena-Giraldo

**Affiliations:** 1 Grupo de Investigación Biología de Sistemas, Escuela de Ciencias de la Salud, Facultad de Medicina, Universidad Pontificia Bolivariana, Medellin 050036, Colombia; maritza.londono@upb.edu.co (M.L.-B.); isabel.ortiz@upb.edu.co (I.C.O.-T.); lina.hoyos@upb.edu.co (L.M.H.-P.); lyorozcoj@yahoo.es (L.Y.O.); 2 Academic Department of Engineering, Pontificia Universidad Católica de Perú, San Miguel 15088, Peru; sm.perez@pucp.edu.pe; 3 Grupo de Investigación en Salud Pública, Escuela de Ciencias de la Salud, Facultad de Medicina, Universidad Pontificia Bolivariana, Medellin 050036, Colombia; lucelly.lopez@upb.edu.co; 4 Immunology and Virology Laboratory, Universidad Autónoma de Nuevo León, Monterrey 64450, Mexico; diana.zaratetr@uanl.edu.mx; 5 Department of Chemistry and Biochemistry, Centre for NanoScience Research, Concordia University, Montreal, QC H4B 1R6, Canada; john.capobianco@concordia.ca

**Keywords:** genotoxicity, cytotoxicity, biocompatibility, viability, cardiomyocytes, fibroblasts, azobenzene, photosensitive polymeric nanocarriers, photoisomerization, drug photorelease

## Abstract

Drug nanoencapsulation increases the availability, pharmacokinetics, and concentration efficiency for therapeutic regimes. Azobenzene light-responsive molecules experience a hydrophobicity change from a polar to an apolar tendency by *trans–cis* photoisomerization upon UV irradiation. Polymeric photoresponse nanoparticles (PPNPs) based on azobenzene compounds and biopolymers such as chitosan derivatives show prospects of photodelivering drugs into cells with accelerated kinetics, enhancing their therapeutic effect. PPNP biocompatibility studies detect the safe concentrations for their administration and reduce the chance of side effects, improving the effectiveness of a potential treatment. Here, we report on a PPNP biocompatibility evaluation of viability and the first genotoxicity study of azobenzene-based PPNPs. Cell line models from human ventricular cardiomyocytes (RL14), as well as mouse fibroblasts (NIH3T3) as proof of concept, were exposed to different concentrations of azobenzene-based PPNPs and their precursors to evaluate the consequences on mitochondrial metabolism (MTT assay), the number of viable cells (trypan blue exclusion test), and deoxyribonucleic acid (DNA) damage (comet assay). Lethal concentrations of 50 (LC50) of the PPNPs and their precursors were higher than the required drug release and synthesis concentrations. The PPNPs affected the cell membrane at concentrations higher than 2 mg/mL, and lower concentrations exhibited lesser damage to cellular genetic material. An azobenzene derivative functionalized with a biopolymer to assemble PPNPs demonstrated biocompatibility with the evaluated cell lines. The PPNPs encapsulated Nile red and dofetilide separately as model and antiarrhythmic drugs, respectively, and delivered upon UV irradiation, proving the phototriggered drug release concept. Biocompatible PPNPs are a promising technology for fast drug release with high cell interaction opening new opportunities for azobenzene biomedical applications.

## 1. Introduction

Various naturally occurring semi- and synthetic polymers make multifunctional nanoparticles (MNPs). They hold the potential to encapsulate peptides, drugs, nucleic acids, and proteins, among others, protecting and increasing their activity [1]. MNP exposition in biological systems may induce toxic responses, especially when interacting with cellular agents such as plasma membrane, organelles, and genetic material [2]. The evaluation of new nanomaterials is required before their uses in real scenarios. In vitro test evaluations provide a quick and effective tool for examining a series of toxicological criteria, such as cellular viability, alterations in metabolism, population growth, and structural alterations, defining nanomaterials’ effects through different concentrations of MNPs in cells [3].

Advances in research have positioned chitosan as a biodegradable and biocompatible nanomaterial to manufacture MNPs oriented to the pharmacological industry [4]. Chitosan with different molecular weights and deacetylation degrees have been functionalized with many molecules to improve or modify their properties [5]. N-succinyl chitosan (NSC) is a water-soluble chitosan derivative with low toxicity and long-term circulation retention [6,7]. Recent investigations have demonstrated the use of NSC as a drug vehicle through functionalization with other components, such as low-density lipoproteins [8], for releasing siRNA and paclitaxel, as well as ion treatment for the release of acid aminosalicylic [9]. These studies have concluded that MNPs based on NSC are compatible with biological systems, and the toxicity of chitosan products depends not only on their physicochemical properties but also on the concentrations in cell studies. Chitosan is a biopolymer with low toxicity, but its derivatives must be carefully evaluated.

External stimulus methods over sensitive nanosystems offer control for fast drug release, such as exposure to radiation [10], temperature alteration [11], and pH change [12], among others. Light-triggered polymeric photoresponse nanoparticles (PPNPs) are an attractive option for cargo release kinetics increase because they have a noninvasive nature and spatiotemporal external control [13]. Introducing light-responsive molecules into a biopolymer backbone is a strategy to give photoresponse ability to nanosystems [14]. Azobenzene derivatives based on 2-[4-phenylazo phenoxy] ethanol (PAPE) are among the most widely developed photoresponsive molecules [15]. They undergo reversible UV-visible photoisomerization of their nitrogen double bond (N=N) [16]. UV irradiation converts apolar *trans*-isomers into polar *cis*-isomers, which can be reconverted to *trans*-isomers by visible light [17]. Phototechnologies use PAPE’s spatiotemporal control and photoisomerization properties as the principal mechanism for bioapplications [18]. Among these standouts are nanosphere [19] and micelles [20] formation for localized cargo release concepts [21], polymeric nanomotors for movement generation and kinetics acceleration [22], potential cancer treatments [23], and photosensitive micromotors for enzymatic activity protection against UV light [24], among others.

**Figure 1:**
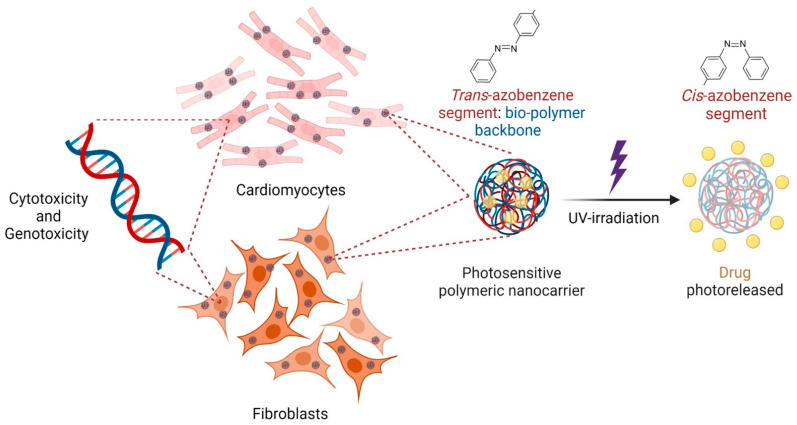
Schematic illustration of cytotoxicity and genotoxicity of azobenzene-based polymeric nanocarriers for phototriggered drug delivery in biomedical applications.

The toxicity evaluation of azobenzene compounds has not been entirely detailed, and its relatively slow degradation rate has been the main obstacle to its use as a drug transporter [25]. To the best of our knowledge, the genotoxicity effects of azobenzene molecules and their derivative polymers have not been studied sufficiently and are highly demanded in developing new phototherapies. This work reports a complete evaluation of the cytotoxic effects and the first genotoxic effects of photosensitive nanocarriers based on an azobenzene derivate (PAPESE) and an NSC biopolymer developed in previous work [26]. Amidation between PAPESE and NSC was employed to synthesize the photosensitive polymer and a nanoprecipitation methodology was used for PPNP self-assembly. As model and antiarrhythmic drugs, Nile red and dofetilide were efficiently encapsulated separately and photodelivered upon UV irradiation by *trans–cis* photoisomerization of the azobenzene contained on the PPNPs. We demonstrated the biocompatibility of PPNPs and the slight toxicity of their precursors, which are essential for phototriggered drug release, as a promising therapy in biomedical applications (Figure 1).

## 2. Materials and Methods

All the reagents were commercially acquired from Sigma-Aldrich (St. Louis, MO, USA). The human fetal ventricular cardiomyocytes, RL14 cells, were obtained from American Type Culture Collection (ATCC, PTA-1499), Ciudad de Mexico, Mexico. The NiH3T3 fibroblasts were donated by the Tissue and Cell Therapy Engineering Group from the University of Antioquia.

### 2.1. Synthesis, Self-Assembly, and Characterization of PPNPs

PPNPs were synthesized and self-assembled using a previously published method [26]. In summary, PAPESE was activated with N,N′-Dicyclohexylcarbodiimide (DCC) and N-Hydroxysuccinimide (NHS) in 10 mL dimethylformamide (DMF) (molar ratio 1:1:2, respectively) for 24 h at 1000 rpm. Activated PAPESE was precipitated with 5 wt.% NaOH, purified by filtration to remove the solvent, and strong-washed with acetone and ethanol. Afterward, 1 mg/mL NSC solubilized in 0.4 M acetic acid and 0.3 mg/mL activated PAPESE in DMF were reacted under constant stirring for 72 h. The photosensitive polymer was precipitated and washed in the same form as the activated PAPESE and dialyzed against water for 24 h.

PPNPs were self-assembled with a nanoprecipitation methodology, where 1 mL tetrahydrofuran (THF) was dripped on a diluted photosensitive polymer in an aqueous solution at pH 5 with a volume ratio of 1:1 at 1 ml/h. After, 1 mL water and THF solution at a volume ratio of 0.9:0.1 was dripped on the last dispersion at the same speed. The THF was evaporated a room temperature for 24 h, and the PPNPs were dialyzed against water for 24 h.

The structure compositions of NSC, PAPESE, and PPNPs, as well as the PPNP morphology, were characterized with Fourier-transform infrared (FT-IR), PerkinElmer, FT-IR spectrometer at Universidad Autonoma de Nuevo León (UANL), México, and transmission electron microscopy (TEM), FEI Tecnai G2 20 electron microscope, LifeScience Microscopy Facility, Purdue University, USA. The nanoparticles’ size and surface charges were obtained using dynamic light-scattering (DLS) and electrophoretic light-scattering (ELS) with a Zetasizer analyzer (Malvern Nano-ZS) at Centro de Investigacion en Materiales Avanzados (CIMAV), Monterrey, Mexico. 

### 2.2. PPNPs Capture by Cells and Characterization

The PPNPs were incubated with RL14 cardiomyocytes and NiH3T3 fibroblasts for 24 h. The supernatant was removed and, after fixation and washing, 1 μg/mL calcein dye was added. Confocal laser microscopy (Axio Observer Z1/LSM 700, Zeiss at UANL, Mexico) and a 60× objective was used for morphological and PPNP–cell interaction analyses. The PPNPs and calcein dye were excited at 2 mV, with 405 and 488 nm lasers, respectively.

### 2.3. Cell Culture

RL14 and NIH3T3 were the selected cellular lines. RL14 cell lines of human fetal ventricular cardiomyocytes have been employed as in vitro models to study the effects of drugs in the heart [27,28]. NIH3T3 mouse fibroblasts are a cell line derived from mouse embryos and were used as a model for nanoparticle-mediated toxicity studies [29].

The cells were maintained in DMEM supplemented with 10% (vol/vol) fetal bovine serum at 37 °C in a humidified atmosphere containing 5% CO_2_. Biocompatibility assays were performed when the cell cultures were at 80% confluence [27].

### 2.4. MTT Assay

An MTT assay was conducted following an established protocol [30,31] to assess the short-term cytotoxic effect of PPNPs on RL14 cardiomyocytes and NiH3T3 fibroblasts. It was employed as an indicator of the cells’ metabolic competence [32]. In summary, RL14 and NiH3T3 cells (4 × 103 cells/well, 96-well microplates) were incubated with 10 μL of 20 freshly prepared serial dilutions (1:2) of NSC, PAPESE, and PPNPs, ranging from 2000 to 3.81 ×10−3 µg/mL, for 24 h. At the end of the incubation, 10 μL MTT solution (5 mg/mL) was added to each well. The microplates were then incubated under constant stirring in the dark for 6 h at 37 °C. Then, 100 μL cold dimethylsulfoxide (DMSO) was added to each well to dissolve the formazan crystals, followed by gentle stirring in a gyratory shaker for between 12 to 24 h. After that, the optical density (OD) was measured with a Multiskan-go spectrophotometer employing a wavelength of 570 nm. Five independent experiments were performed in triplicate to verify the repeatability. Positive, DMSO 100%, and negative complete culture media without cell controls were implemented.

### 2.5. Trypan Blue Exclusion Assay

The RL14 cells (5 × 104 cells/well, 6-well microplates) were incubated with NSC, PAPESE, and PPNPs (2, 1, 0.5, 0.25, and 0.125 mg/mL) following the Strober protocol [33]. After 24 h of incubation, the cells were trypsinized and counted after adding 0.4% trypan blue vital dye (1:1) using a Newbauer chamber. Three independent experiments were performed on different days in triplicate.

Death probability was performed using the probit analysis program (IBM Corp. IBM SPSS Statistics for Windows, version 19.0. Armonk, New York, USA), which estimates the probability of death at higher treatment concentrations, the lethal concentration of 50 (LC50), and the lethal concentration of 10 (LC10). Statistical analyses were used to compare the extent of dead cells between treatments and concentrations. Significant differences were considered with *p* < 0.05.

### 2.6. Comet Assay

Fifty thousand human ventricular cardiomyocytes (RL14) were seeded per well in a 6-well plate and analyzed with the sublethal concentrations of the different treatments (concentrations less than or equal to LC10) for 24 h. Samples of untreated cells and positive controls of hydrogen peroxide (100 µM) were included in the experiment, and DMEM culture medium was used as a negative control. The six-well plates were washed twice with 0.9% saline solution after 24 h of treatment. They were peeled off and centrifuged at 1200 rpm for 7 min. The trypan blue dye exclusion test was then applied to ensure that the cell viability was more significant than 85%. The individual cells were embedded in low-molecular-weight gelling agarose, and the samples were prepared. After 30 min, the cells were lysed using lysis solution, 0.26 M NaOH, 1.2 M sodium chloride, 0.1% N-lauroyl sarcosine, and 100 Mm EDTA for 24 h. The cells were then washed with an alkaline solution, 0.10 M NaOH, and 2 mM EDTA with a pH greater than 13 and were subjected to alkaline gel electrophoresis. Subsequently, the staining was performed with 2.5 μg/mL ethidium bromide (BrEt) and analyzed using a fluorescence microscope with 40× magnification and considering the tail length. Fifty cells were analyzed in total for each treatment.

Once the comet length data were obtained, damage classification was performed considering genetic damage from the median of the negative control plus one standard deviation (Table 1). The maximum comet length was assumed, and the minimum damage was subtracted. The results were divided into four categories: no, low, medium, and total damage [34].

Subsequently, a damage-weighted index (WDI) was calculated using Equation (1). For each of the treatments evaluated, the cell extent in each of these categories was calculated. Zero damage was obtained from the negative control (Equation (1)):

WDI = n_1_ + 2n_2_ + 3n_3_ + 4n_4_ (1)

where n_1_ is the number of cells with level one damage, n_2_ is the number of cells with level two damage, n_3_ is the number of cells with level three damage, and n_4_ is the number of cells with level four damage.

### 2.7. PPNP Photoisomerization, Drug Nanoencapasulation, and Photorelease

A PPNP concentration was dispersed in DMF and exposed to different times of UV irradiation, and a Multiskan-go spectrophotometer measured the UV-vis spectra.

Each drug was nanoencapsulated in the PPNP self-assembly process, in which excess amounts of Nile red and dofetilide were solubilized in a THF solution, dripped on an aqueous photosensitive polymer solution, and finished as previously described in Section 2.1. The PPNPs were centrifuged to determine the load capacity indirectly, quantifying the drug in the supernatant using spectrophotometry. Then, dialysis was used to purify the drug-loaded PPNPs.

The drug-loaded 0.16 mg/mL PPNPs were dispersed in DMSO, and UV irradiation photodelivered the drug. Finally, the dispersion was filtered to remove the PPNPs, and the spectrophotometer measured the drug-delivered UV-vis spectra, tracking their UV absorption characteristic peaks.

## 3. Results

### 3.1. Photosensitive Nanoparticle Characterization

**Figure 2:**
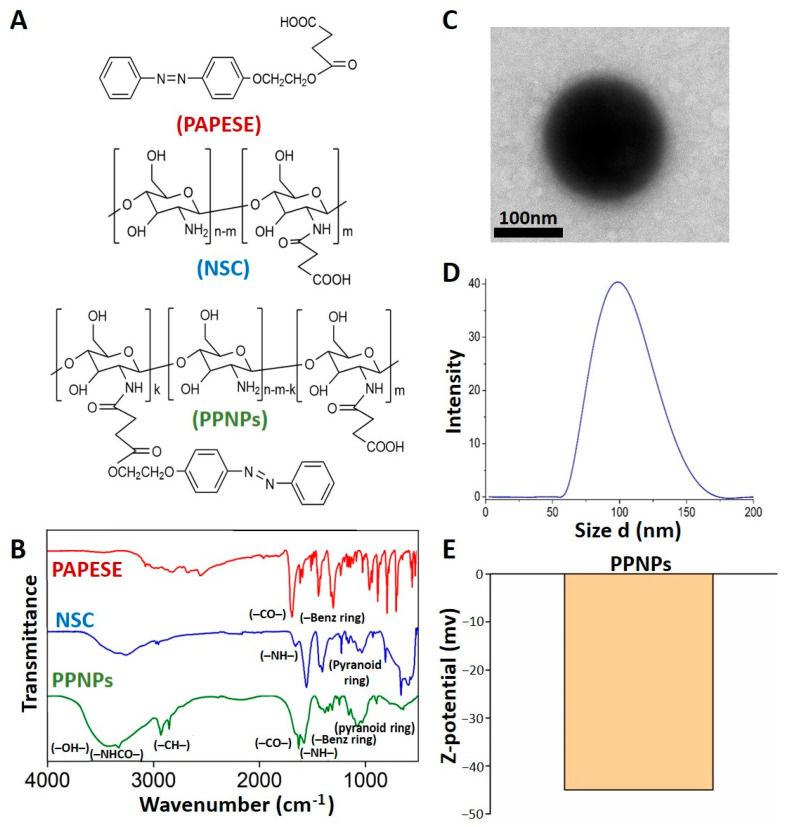
Chemical structures (**A**) and FT-IR characterizations (**B**) of evaluated treatments, the polymeric photoresponse nanoparticles (PPNPs), and their precursors, azobenzene derivate (PAPESE), and N-succinyl chitosan (NSC). TEM image (**C**), hydrodynamic particle size distribution in distilled water (**D**), and Z-potential graphic (**E**) of PPNPs.

The evaluated treatments corresponding to the PPNPs and their precursors (Figure 2A) were adequately characterized by FT-IR (Figure 2B). Likewise, the morphologies, sizes, and surface charges of the PPNPs were characterized with TEM, DLS, and ELS, respectively (Figure 2C–E).

#### 3.1.1. FT-IR Detailed Spectral Data of the PPNP Synthesis

PAPESE: IR (KBr) (Figure 2B-PAPESE): 3069 cm^−1^ (phenyl group, str), 2844, 2805 cm^−1^ (C–H, str), 1682 cm^−1^(–CO–, str), 1599, 1582, and 1494 cm^−1^ (benzene ring, str).

NSC: IR (KBr) (Figure 2B–NSC): 3254 cm^−1^ (–OH, str), 2971, 2945 cm^−1^ (–CH–, ext), 1655 cm^−1^ (–CO–NH–, str), 1553 cm^−1^ (–NH–, bending), 1155 cm^−1^ (–C–O–C–, str), 1024 cm^−1^ (–C–O–, str), and 923 (pyranoid ring, str).

PPNPs: IR (KBr) (Figure 2B-PPNPs): 3472 cm^−1^ (–OH, str), 3323 cm^−1^ (–NHCO–, str), 2923, 2845 cm^−1^ (C–H, ext), 1623 cm^−1^ (–C–O–, str), 1571 cm^−1^ (–NH–, bending), 1463, 1432, 1414 cm^−1^ (benzene ring, str), 1241 cm^−1^ (–C–O–C–, str), 1085 cm^−1^ (–CO–, str), and 846 cm^−1^ (pyranoid ring str).

#### 3.1.2. Morphologies, Sizes, and Surface Charges of the PPNPs

The TEM image (Figure 2C) displays the spherical nanocarrier morphology and confirmed their diameters as around 100 nm. DLS with an average diameter of 98.63 nm (Figure 2D) determined the hydrodynamic size distribution of nanoparticles dispersed in water. In the same conditions, ELS quantified the PPNP surface charges at −45 mv (Figure 2E).

### 3.2. Nanoparticle Catchment by Cells

**Figure 3:**
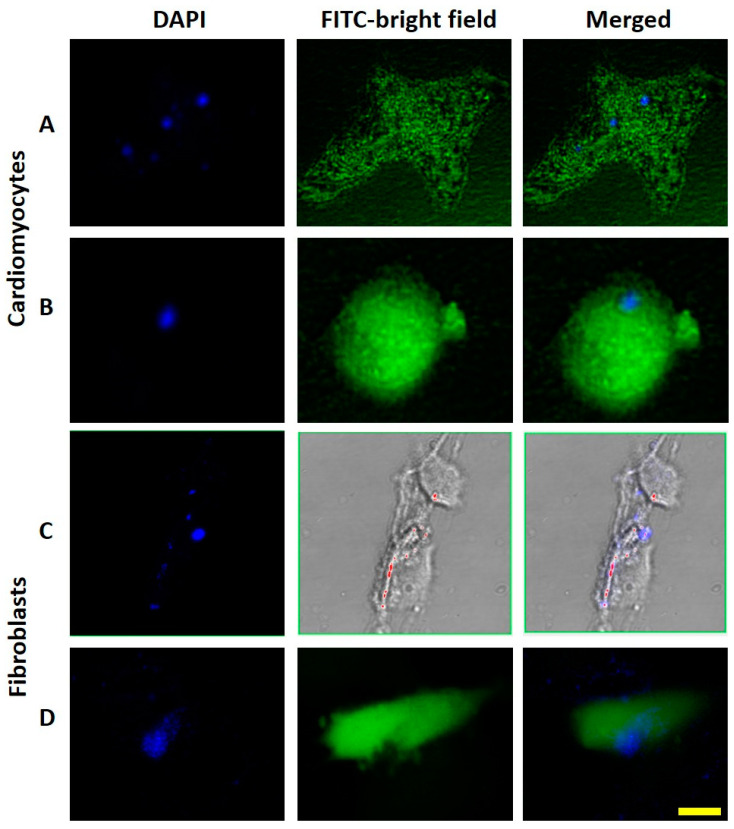
Confocal microscopy images of self-fluorescent and agglomerated PPNPs (observed with a DAPI filter), the cell (stained with calcein using a FITC filter and a bright field filter, respectively), and both together (merged). The PPNPs captured in the cardiomyocytes (**A**,**B**) and fibroblasts (**C**,**D**) upon incubation time of 24 h. The yellow scale is 25 μm.

The confocal images (Figure 3) reveal the self-fluorescence and agglomerated PPNPs in blue, as well as the morphologies, cytoplasm, and nuclei of the cells in green. An amount of 24 h of incubation time demonstrated the correct PPNPs captured by the cells. Cardiomyocytes both without and with stress were able to capture the PPNPs (Figure 3A,B, respectively). Similarly, the fibroblasts produced a complete catchment of the PPNPs, proved in both bright and dark fields (Figure 3C,D, respectively).

### 3.3. Evaluation of Mitochondrial Metabolism

**Figure 4:**
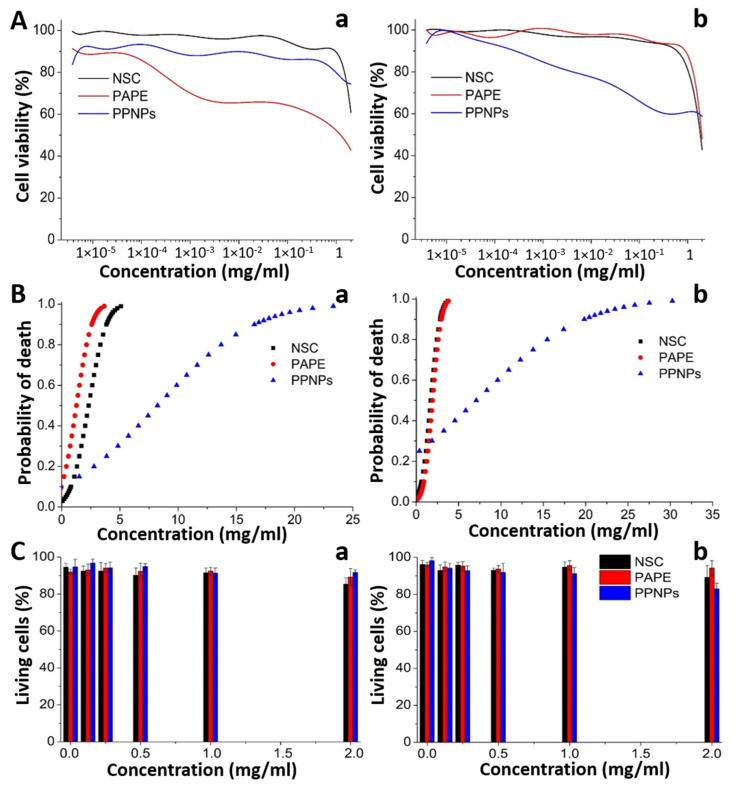
Concentration response curve for cytotoxicity assay (**A**), death probabilities (**B**), and percentage of living cells with DMEM culture medium as a negative control (*p* < 0.001) (**C**) of exposed RL14 cardiomyocytes (**a**) and NiH3T3 fibroblasts (**b**) performed after 24 h using NSC, PAPESE, and PPNP treatments, respectively.

The cytotoxicity induced by PPNPs (Figure 2A-PPNPs, and C), PAPESE (Figure 2A-PAPESE), and NSC (Figure 2A-NSC) was evaluated after treating cardiomyocytes and fibroblasts with 20 successive dilutions ranging from 2000 to 3.81 × 10^−3^ µg/mL during 24 h. The cell survival response of RL14 cardiomyocytes and NiH3T3 fibroblasts to the treatments was dose-dependent, as revealed in Figure 4a,b, respectively.

The photoresponsive moiety (PAPESE) and the polymeric backbone (NSC) precursors induced higher toxicity than the PPNPs. The structure of the nanocarriers led to more outstanding biocompatibility than the precursors since the most toxic molecules were inside the hydrophobic core, minimizing their contact with the cells. A Probit analysis determined the lethal concentration 50 (LC50) of PPNPs and their precursors for cardiomyocytes and fibroblasts. A higher PPNP concentration was required to achieve the same effect as the precursors, and their synthesis concentrations were lower than the LC50 (Table 2).

The NSC and PAPESE precursors presented an antagonistic effect after using statistical correlation to estimate the death probability of the cardiac cells, in which the PPNPs generated less toxicity than their precursors (Figure 4(Ba,Bb)). Exposure to RL14 cardiomyocytes and NiH3T3 fibroblasts at concentrations close to 5 mg/mL caused 100% mortality for the cell population. The viability of cells treated with PPNPs at the same concentration decreased but were outlying from the total cell death.

### 3.4. Evaluation of Membrane Stability

A trypan blue test evaluated the membrane stability of RL14 and NiH3T3 exposed to PPNPs and their precursors at concentrations between 0.13 and 2.00 mg/mL. The test was associated with cell viability, and the results were compared with the negative control, cells exposed to DMEM. NSC did not affect the cell membranes of the two cell lines exposed at any of the concentrations tested (Figure 4(Ca,Cb)). PAPESE only significantly affected the cardiomyocytes (*p* = 0.0002) at a 2.00 mg/mL concentration, decreasing cellular viability by 20%. Nevertheless, these concentrations did not affect the viability of the fibroblasts. The PPNPs only significantly reduced (*p* = 0.00018) the fibroblast viability by 20%.

### 3.5. Genotoxicity Test

**Figure 5:**
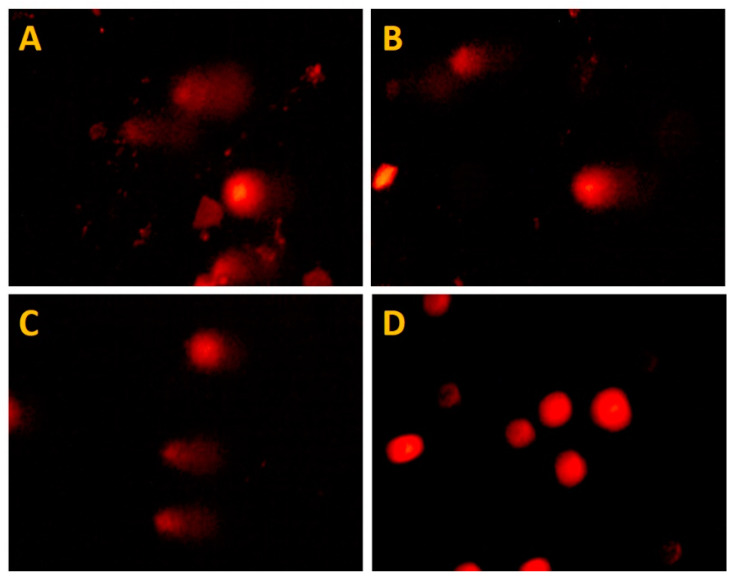
Induced comets by exposure of RL14 cardiomyocytes to the treatments: PPNPs (**A**), NSC (**B**), PAPESE (**C**), and negative control (**D**).

Comets generated by cardiomyocyte exposure to the PPNPs and their precursors are presented in Figure 5, as well as their measurements in Table 3.

**Figure 6:**
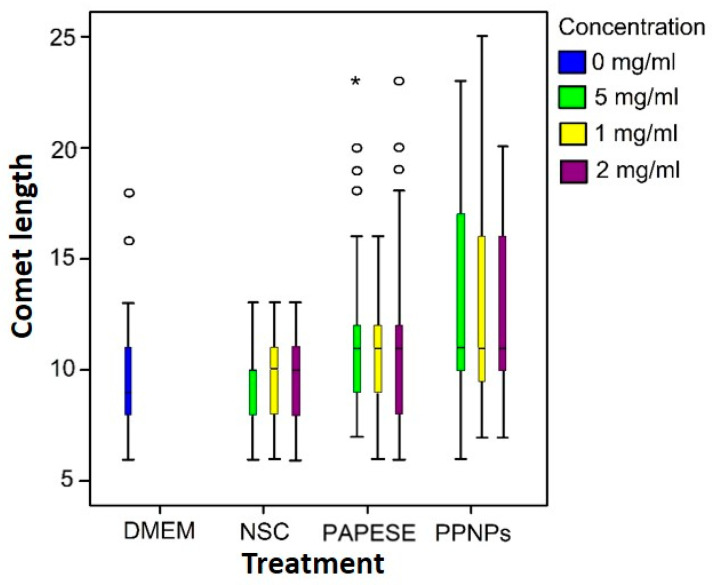
Comet length of human RL14 ventricular cardiomyocytes exposed to PPNPs and their precursors for 24 h. DMEM culture medium was used as a negative control. Outliers circles, and * *p* < 0.001.

As revealed by the migration of deoxyribonucleic acid (DNA) and reflected in the comet size, an analysis of the data suggested that all the treatments induced DNA damage (Figure 6). There were significant differences between the comets generated by the treatments and the negative control (*p* = 0.043 for NSC and *p* < 0.001 for both PAPESE and PPNPs).

Once the damage categories were defined (Table 1), each comet length measured was placed in a corresponding damage category (Table 4). Although the results revealed that nanoparticles and their precursors at 2 mg/mL and 0.5 mg/mL induced DNA damage, more than 50% of the cell population was undamaged. A proportion lower than 20% presented medium damage, and no cell presented total (or high) damage to the genetic material. In addition, no dependence on genotoxicity was found with the concentrations of the precursors and PPNPs. Finally, the weighted damage index for each treatment and concentration was calculated using Equation (1) (Table 4).

### 3.6. PPNP Photoisomerization and Drug Photorealase

**Figure 7:**
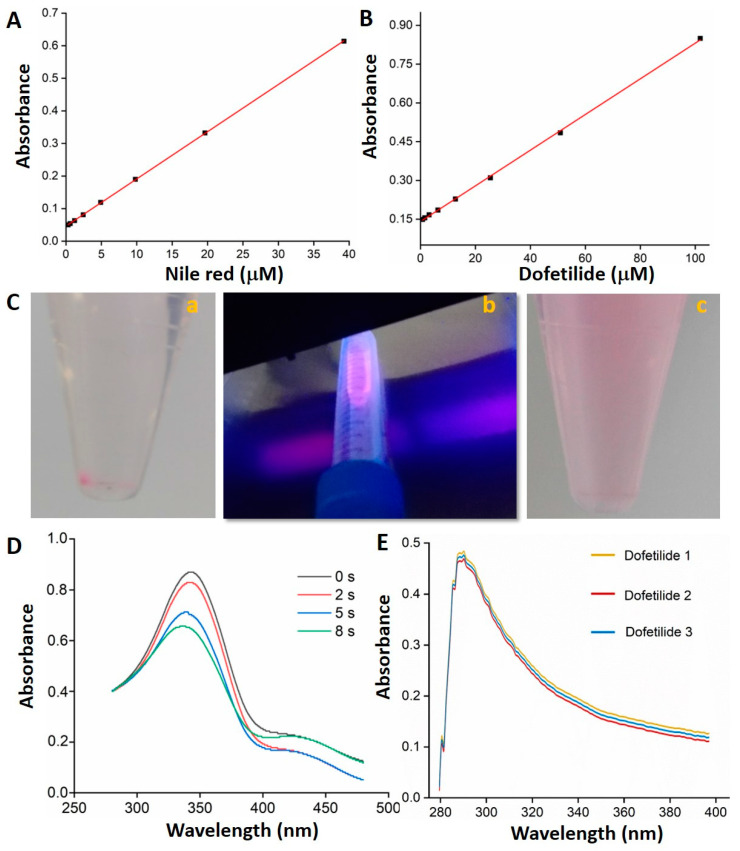
Calibration curves of Nile red (**A**) and dofetilide (**B**) in DMSO. Nile red photorelease process (**C**). Pictures of Nile red encapsulated by PPNPs centrifuged (**a**), UV irradiated (**b**), and photodelivered (**c**). UV-vis spectra variation of azobenzene-based polymeric nanocarriers by *trans–cis* photoisomerization at different UV irradiation times (**D**). UV spectra of photodelivered dofetilide (**E**).

The behavior of the UV absorption drugs concerning their concentrations in DMSO was presented using Nile red and dofetilide (Figure 7A,B). The encapsulation, photostimulation, and release of Nile red are illustrated in Figure 7(Ca–Cc), in which the centrifuged, loaded drug was released by the *trans–cis* photoisomerization of the azobenzene-based PPNPs, characterized by a decrease in the 343 nm peak of the UV-vis spectra upon UV irradiation (Figure 7D). In the same way, dofetilide was photodelivered and tracked using the UV-vis spectra (Figure 7E).

Linear Equations (2) and (3) respectively express the concentration absorbance curves from Figure 7A,B, following the peaks at 288 nm and 555 nm ranging from 0.307 to 39.26 μM and 0.7957 to 101.85 μM with correlation coefficients (R^2^) of 0.99996 and 0.99964 for Nile red and dofetilide.

(2) $$\mathrm{Nile}\;\mathrm{red}\;\left(µM\right)=\frac{\mathrm{Abs}-0.04618}{0.01449}$$

(3) $$\mathrm{Dofetilide}\;\left(µM\right)=\frac{\mathrm{Abs}-0.1409}{0.0069}$$

Equations (2) and (3) were employed to quantify the loaded and delivered drug per PPNP concentration after UV irradiation (Table 5).

## 4. Discussion

Toxicological evaluation precedes nanoparticle development in biomedicine to guarantee their safety or define their potential danger [3]. Moreover, azobenzene has a high potential for application in nanocarrier technologies. A biocompatibility analysis was proved in this context, evaluating the viability and genotoxicity of azobenzene-based PPNPs for opening the possibility of new photosensitive therapies.

The PPNPs were synthesized through an amidation reaction using the DCC activating agent of carboxylic groups from PAPESE to link with free amines from NSC covalently. The product was verified with FT-IR, in which the amide characteristic peak appeared at 3323 cm^−1^, the benzene and pyranoid ring representative peaks from PAPESE and NSC remained, and the carbonyl peak shift from 1682 cm^−1^ to 1623 cm^−1^, corresponding to the carboxyl from PAPESE and producing PPNP terminal groups (Figure 2A,B). The photosensitive amphiphilic polymers and their nanocarrier assembly (Figure 2C) displayed the correct solid sphere structure formation with a micelle tendency. The azobenzene derivative was the hydrophobic segment of the polymer, and the carboxylic group from NSC was the hydrophilic one. A nanoprecipitation method using organic solvent evaporation oriented and agglomerated the azobenzene molecules to the PPNP core, with the ability to encapsulate hydrophobic cargo. The carboxylic groups were extended to the surface, presenting a −45 mv negative charge (Figure 2E). The azobenzene group was compacted into the nanocarriers, permitting blue self-fluorescence and tracking to prove their cell capture by the cardiomyocytes and fibroblasts (Figure 3), which was possible thanks to their nanosize (Figure 2D), allowing a cell viability study of the cytotoxicity and genotoxicity effects.

The evaluation of mitochondrial metabolism stability through an MTT assay displayed that exposure promoted mitochondrial dysfunction in a dose-dependent way; the cell viability decreased as the concentrations of the PPNPs and precursors increased. In addition, a projection using a Probit analysis, generated from the readings of the optical densities and their relationships with cell proliferation allowed the discovery of the LC50 for each evaluated treatment. This work presented low cytotoxic effects of NSC in concentrations up to 2 mg/mL on human RL14 cardiomyocytes (LC50 of 2.09 mg/mL) and mouse NiH3T3 fibroblasts (LC50 of 1.28 mg/mL). NSC had shown no significant effects on cell viability at concentrations between 0 and 0.25 mg/mL for the NiH3T3 fibroblast cell line. We included a novel cytotoxicity evaluation of the PAPESE molecule, determining a value of 1.72 mg/mL for NiH3T3 fibroblasts and 1.28 mg/mL for RL14 cardiomyocytes. The LC50 values were higher than the concentrations required for PPNP synthesis (i.e., 1 mg/mL of NSC and 0.5 mg/mL of PAPESE). The LC50 of the PPNPs was higher than 3 mg/mL, which was above the concentration required for a high internalization of PPNPs into the cardiac cells checked using previously reported work (0.16 mg/mL) [26], demonstrating that the PPNP concentration necessary to transport the dofetilide antiarrhythmic drug inside cardiac cells did not present cytotoxicity.

The PPNPs had a more negligible effect on cellular viability than the precursors. The PPNPs presented spherical structures, with their carboxylic acids exposed on the surfaces, and their PAPESE segment was oriented to the nucleus. PAPESE induced a toxic effect, but the cellular viability increased when the biopolymer based on Chitosan covered it, which is equivalent to reported works in which Chitosan nanomatrices were not toxic at concentrations lower than 1 mg/mL [35]. Trypan blue dye in the presence of PPNPs determined the cell membrane stability. NSC at concentrations between 0.13 and 2 mg/mL did not affect the cell membranes of cardiomyocytes and fibroblasts, consistent with the literature [36,37]. PAPESE had no adverse effects on fibroblast viability, but the cardiomyocyte viability decreased at 2 mg/mL. The stability evaluation of the cardiomyocyte and fibroblast membranes demonstrated that the PPNPs caused cell membrane damage at 2 mg/mL, but not at lower concentrations. These outcomes suggested that the azobenzene-based polymer degradation rate is relatively slow, benefiting PPNP stability and viability.

This work highlighted the first genotoxic study of azobenzene-based PPNPs and PAPESE molecules in cardiomyocytes compared to similar reported studies [35,38]. An alkaline comet assay evaluated the effects on human cardiomyocytes DNA. NSC and PAPESE precursors, as well as PPNPs at concentrations between 0.5 mg/mL and 2.0 mg/mL, generated low and medium levels of DNA damage, reflected by an increase in the comet tail length. Nevertheless, the mechanism of DNA damage is still unclear [39]. Several causes can produce cell damage, such as direct material–DNA interaction, indirect oxidative stress, and the generation of reactive oxygen species (ROS) [3,40]. Excessive ROS production possibly surpassed the cells’ antioxidant capacity, producing alterations in the mitochondrial metabolism, as observed in the MTT test. The ROS could interact with the cell membrane, delivering lipid peroxidation and, thereby, leading to cell death or oxidative stress that could induce DNA damage.

Accordingly, the cytotoxic and genotoxic effects determined that less than 3 and 2 mg/mL of PPNPs presented viability to the evaluated cell lines. An amount of 0.16 mg/mL PPNP biocompatible concentration encapsulated 19.38 + 0.07 and 57.52 ± 0.09 μM, and photoreleased 98.71 and 97.12% of the Nile red and dofetilide hydrophobic drugs, respectively (Figure 7 and Table 5), upon UV irradiation exposure (Figure 7(Cb,E)) by azobenzene-based PPNP *trans–cis* photoisomerization (Figure 7D). The delivered dofetilide antiarrhythmic drug depicted a therapeutic concentration with release-accelerated kinetics for possible atrial fibrillation treatment, as established in a previously reported work (5 μM of dofetilide) [41]. Then, lower PPNP concentrations were safe and valuable for biomedical applications such as drug delivery, intracellular diagnosis, photodynamic therapy, and theragnosis, among others.

## 5. Conclusions

The cytotoxicity and genotoxicity was determined for the cellular viability of human RL14 cardiomyocytes and mouse NiH3T3 fibroblasts with PPNP concentrations lower than 3 and 2 mg/mL through membrane damage with a trypan blue test, metabolism alteration with an MTT test, and DNA damage with a comet assay.

PAPESE, NSC, and PPNP treatments caused low or medium damage to the genetic material, but the proportion of affected cells did not exceed 50%, and none presented severe or total DNA damage, indicating that the azobenzene-based PPNPs were biocompatible.

It is highly recommended to evaluate whether there are cell-cycle alteration or mutagenic effects, to define whether the degree of PPNP compaction has any effect, and to evaluate whether oxidative stress and ROS generation produce cell affectations.

A biocompatible PPNP concentration of 0.16 mg/mL photodelivered 98.71 and 97.12% of Nile red and dofetilide drugs, respectively, with potential therapeutic concentration through *trans–cis* photoisomerization upon UV exposure.

The azobenzene-based polymeric nanocarriers were postulated as a potential candidate for a biocompatible, accelerated drug release system in biological tissue with a therapeutic regime. They close the gap between technologies that use azobenzene for its cytotoxic effect, which are highly applied in cancer therapies.

## Figures and Tables

**Table 1:** Deoxyribonucleic acid (DNA) damage levels of cells exposed to different concentrations of polymeric photoresponse nanoparticles (PPNPs) and their precursors.

Damage Level	Damage Category	Tail Length (µm)
0	No damage	0–11.34
1	Low damage	11.34–17.34
2	Medium damage	17.34–23.34
3	Total damage	23.34–34.68

**Table 2:** Lethal concentration 50 for RL14 human cardiomyocytes and NiH3T3 murine fibroblasts exposed to PPNPs and their precursors, N-succinyl chitosan (NSC) and azobenzene derivate (PAPESE).

Assay	NSC (mg/mL)	PAPESE (mg/mL)	PPNPs (mg/mL)
PPNPs synthesis	1.00	0.30	
LC50 of RL14 Cardiomyocytes	2.09	1.28	3.80
LC50 of NiH3T3 Fibroblasts	1.28	1.72	3.41

**Table 3:** Comet tail length of human ventricular cardiomyocytes exposed to PAPESE, NSC, and PPNPs.

Treatment	Concentration (mg/mL)	Comet Tail Length (µm)		
		Median	25th Percentile	95th Percentile
DMEM	0	9	8.0	13
NSC	0.5	10	8.0	13
	1.0	10	8.0	13
	2.0	10	8.0	13
PAPESE	0.5	11	9.0	17
	1.0	11	9.0	16
	2.0	11	8.0	16
PPNPs	0.0	11	10.0	20
	1.0	11	9.5	19
	2.0	11	10.0	20

**Table 4:** Percentage of cells found in each damage category and weighted damage index (WDI).

Treatment	Concentration (mg/mL)	Damage Level (% of Cells)				WDI
		0	1	2	3	
DMEM	0	99.1	0.89	0	0	9
NSC	2.0	100.0	0	0	0	0
	1.0	100.0	0	0	0	0
	0.5	100.0	0	0	0	0
PAPESE	2.0	89.3	10.6	0	0	32
	1.0	92.3	7.6	0	0	23
	0.5	89.3	10.6	0	0	32
PPNPs	2.0	74.6	25.3	0	0	76
	1.0	69.0	31.0	0	0	93
	0.5	65.6	34.3	0	0	103

**Table 5:** Concentrations and percentages of drugs encapsulated and photoreleased in DMSO by UV irradiation of PPNPs.

Drug	PPNPs (mg/mL)	Drug Encapsulated (μM)	Drug Photoreleased (%)
Nile red	0.1623 ± 0.0003	19.38 + 0.07	98.71
Dofetilide	0.1614 ± 0.0002	57.52 ± 0.09	97.12

## Data Availability

The datasets used and analyzed during this study are available from the corresponding author upon reasonable request.
